# The value analysis of tumor deposit in the comprehensive evaluation before differentiated thyroid cancer RAI therapy

**DOI:** 10.3389/fonc.2025.1593603

**Published:** 2025-06-02

**Authors:** Lei Wang, Jing Yang, Yuan Zhu, Zhiyong Li

**Affiliations:** Department of Nuclear Medicine, The Affiliated Hospital of Xuzhou Medical University, Xuzhou, China

**Keywords:** tumor deposit, prognosis, differentiated thyroid cancer, radioactive iodine, TNM staging, the ATA initial risk stratification system

## Abstract

**Objective:**

This study aimed to investigate the impact of tumor deposit (TD) on Radioiodine (RAI) therapy efficacy in Differentiated Thyroid Cancer (DTC) and explore their potential role in postoperative staging and the American Thyroid Association (ATA) initial risk stratification system before RAI treatment.

**Methods:**

This study retrospectively analyzed data from a total of 11,278 thyroid cancer surgical patients between 2019 and 2023. Among 2,162 patients were considered eligible for prognostic analysis (2056 cases in the TD-negative group, 106 cases in the TD-positive group). A 1:1 propensity score matching was conducted for the TD-positive group. Single-factor and multiple-factor analyses of prognostic factors were performed for both groups. The predictive abilities of different N stages, TNM staging, and the ATA initial risk stratification system including TD were evaluated.

**Results:**

There were 235 cases (2.08%) of TD-positive patients. Multivariate analysis demonstrated that gender, presence of TD, the ATA initial risk stratification system, and TNM staging were independent prognostic factors. In all patients, those in the N1a stage, TNM stage I, and ATA intermediate risk group, the cumulative incidence of ER in the TD-positive group was lower than that in the TD-negative group (48.6% vs. 77.8%, 37.5% vs. 78.7%, 35.4% vs. 67.4%; P = 0.019, 0.001, 0.013 respectively). Patients with TD-positive in N1a stage had no significant difference in prognosis compared to TD-negative patients in N1b stage (32.6% vs. 42.6%, P = 0.867); TD-positive patients in TNM stage I had similar prognosis to TD-negative patients in TNM stage II (37.5% vs. 28.9%, P = 0.338); TD-positive patients in the ATA intermediate risk group showed no significant difference in prognosis compared to TD-negative patients in the ATA high risk group (35.4% vs. 12.5%, P = 0.300).

**Conclusion:**

TD should be considered as a prognostic factor in the postoperative RAI treatment. We propose incorporating TD into the TNM staging and the ATA initial risk stratification system as a reference, suggesting that TD-positive patients in N1a stage be classified as N1b stage; patients in TNM stage I be classified as TNM stage II ;and the ATA intermediate risk patients be classified as the ATA high risk.

## Introduction

The most common pathological type of thyroid cancer is Differentiated thyroid cancer (DTC), which is characterized by a relatively low disease-specific mortality rate and excellent overall survival (1). Postoperative radioactive iodine (RAI) therapy is one of the main measures for comprehensive management of DTC patients, and a comprehensive assessment before treatment is essential for implementing appropriate therapy. In 2009, the American Thyroid Association (ATA) guidelines classified the risk of recurrence in DTC patients into low risk, intermediate risk, and high risk based on clinical and pathological characteristics at initial treatment, providing important guidance for clinical decision-making (2). Furthermore, postoperative staging helps provide prognostic information for patients to guide individualized treatment strategies and disease monitoring plans. Currently, the most commonly used postoperative staging system for DTC is the 8th edition TNM staging developed jointly by the American Joint Committee on Cancer (AJCC) and the Union for International Cancer Control (UICC) (3).

Tumor deposit (TD) was identified in the pericolonic adipose tissue of colorectal cancer patients and were presumed to represent metastatic nodules within the pericolonic adipose tissue or adjacent mesentery. (4). Since the 7th edition of the TNM staging system, TD has been included in the N stage of colorectal cancer. Any pT lesion lacking regional lymph node metastasis but containing TD is classified as pN1c disease (5). Numerous studies have reported the relationship between the presence of TD and poor prognosis in colorectal cancer (6, 7).

However, the presence and prognostic significance of TD in DTC have not been widely studied. To date, only a small number of retrospective studies in the field of DTC have mentioned TD, with researchers suggesting an association between TD and the prognosis of thyroid cancer or level V lymph node metastasis (LNM) (8, 9). The purpose of this study was to explore the clinical and pathological characteristics of TD, evaluate the prognostic impact of TD on postoperative RAI treatment in patients with DTC, compare the prognosis of TD in different TNM stages and the ATA initial risk stratification system. Based on this, we propose optimized clinical staging and the ATA initial risk stratification system recommendations that included TD as an important consideration factor.

## Patients and methods

### Study population

**Figure 1:**
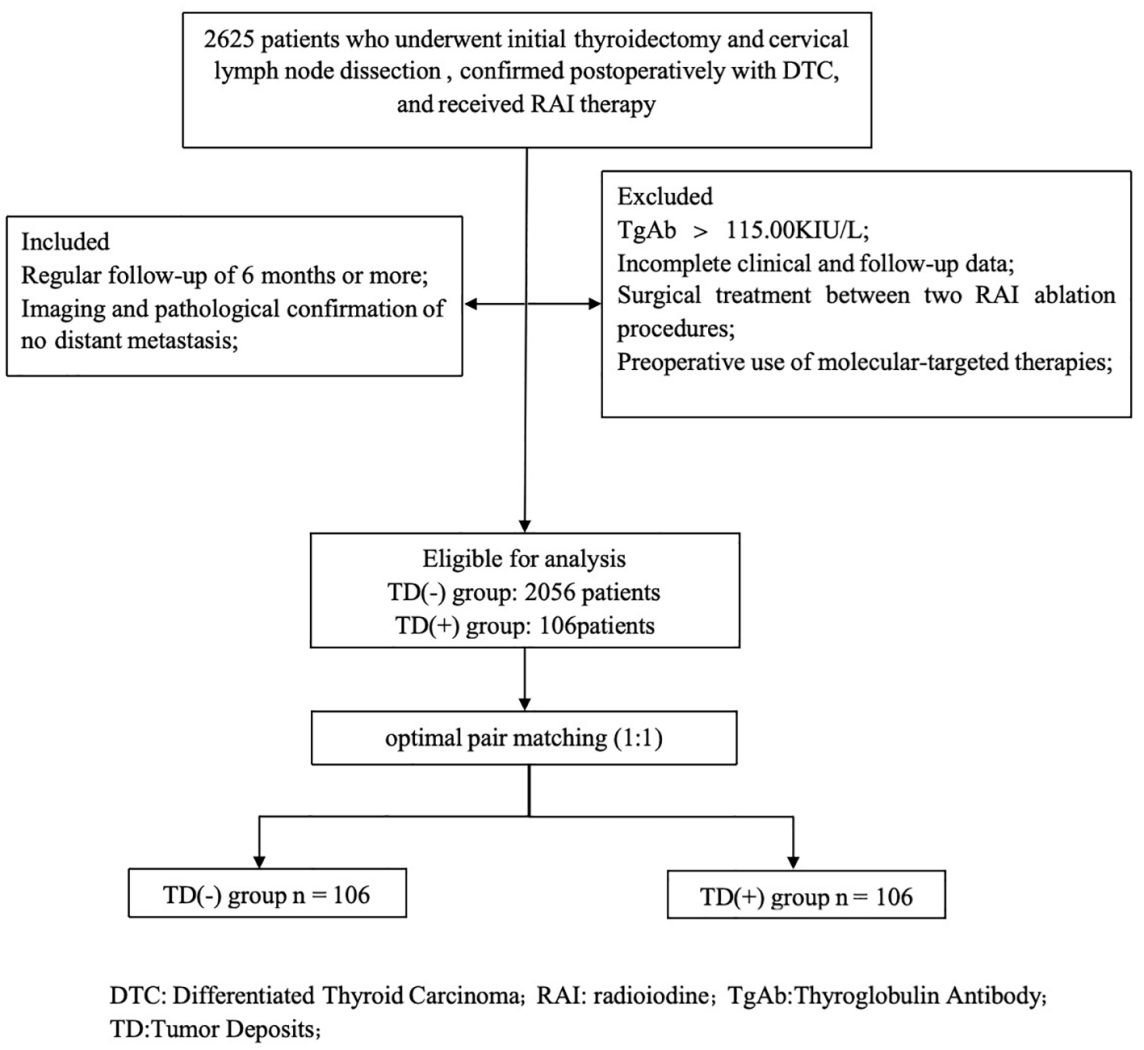
Flowchart of inclusion and exclusion criteria.

A total of 2625 patients who underwent thyroid surgery and neck lymph node dissection at the Affiliated Hospital of Xuzhou Medical University from January 2019 to December 2023, with postoperative pathological confirmation of DTC (including papillary thyroid carcinoma and follicular thyroid carcinoma) and at least one RAI treatment in our department. Inclusion criteria were as follows (1): Regular follow-up of 6 months or more; (2) Imaging and pathological confirmation of no distant metastasis. Exclusion criteria were as follows: (1) Thyroglobulin antibody (TgAb) > 115.00 KIU/L; (2) Incomplete clinical and follow-up data; (3) Surgical treatment between two RAI ablation procedures; (4) Preoperative use of molecular-targeted therapies, including multi-kinase inhibitors (tyrosine kinase inhibitors) or highly selective single-target inhibitors before RAI ablation (**Figure 1**). A total of 2162 patients were finally included. According to the 2015 ATA guidelines (10), the patients achieved the goal of thyroid stimulating hormone (TSH) > 30 mU/L after Levothyroxine withdrawal and followed a low-iodine diet for 3–4 weeks. And cervical lymph nodes ultrasound, chest CT, and other related examinations were also performed. The initial treatment dose for radioactive iodine (RAI) therapy ranges from 1.11 GBq to 5.55 GBq. The whole-body scan (Rx-WBS) and single-photon emission computed tomography/computed tomography (SPECT/CT) were performed within 5 days after RAI therapy. The study was approved by the Medical Ethics Committee of the Affiliated Hospital of Xuzhou Medical University.

### Follow-up data

Dynamic evaluation was conducted every 6 months, involved both serological and imaging measurements, including serum thyroglobulin levels, TgAb, thyroid function especially TSH level, and neck ultrasound. RAI diagnostic scanning (Dx-WBS), chest CT scan or ^18^F-FDG positron emission tomography (PET)-CT scanning were also used if necessary. The subjects were classified based on their post-treatment response as follows: 1. Excellent response (ER): negative imaging and either suppressed Tg < 0.2 ng/mL or TSH-stimulated Tg < 1 ng/mL. 2. Biochemical incomplete response (BIR): negative imaging and suppressed Tg ≥ 1 ng/mL or stimulated Tg ≥ 10 ng/mL or rising anti-Tg antibody levels. 3.Structural incomplete response (SIR): structural or functional evidence of disease with any Tg level with or without anti-Tg antibodies. 4. Indeterminate response (IDR): nonspecific findings on imaging studies; faint uptake in thyroid bed on RAI scanning; nonstimulated Tg detectable, but < 1 ng/mL; stimulated Tg detectable, but < 10 ng/mL or anti-Tg antibodies stable or declining in the absence of structural or functional disease. The final treatment response after RAI therapy was categorized as follows: the ER group and the NER group (including IDR, SIR, and BIR), and the regression of both groups was analyzed. The evaluation period ends when the first evaluation reaches the NER state or when reaching the end of the follow-up deadline. The follow-up for all patients included in this study concluded in September 2024.

### Definition of TD

Referring to the definition in the 8th edition of the AJCC Tumor TNM Staging criteria, TD is defined as discrete tumor nodules of any shape, contour or size that lack associated lymph node tissue, vascular structures or neural structures, found within the lymph drainage area of the primary tumor (3). In this study, any nodule with identifiable blood vessels or lymphatic vessels was classified as vascular or lymphatic invasion; any nodule with identifiable nerve structures was classified as perineural invasion.

### Grouping and staging

When exploring the clinical and pathological characteristics of TD and its impact on RAI treatment, the study divided patients into TD-positive and TD-negative groups. When comparing the prognosis of TD in different N stages, TNM stages, and the ATA initial risk stratification system, the groups were further divided into TD-positive and TD-negative groups in N0, N1a, N1b stages; TNM I, II stages (since there was only 1 case in TNM III stage in this study, no grouping study was conducted); and low, intermediate, high-risk groups. Considering the new staging, the groups were divided into TD(+) N1a group, TD(+) TNM I group, and TD(+) intermediate-risk group.

### Statistical analysis

Analyses were conducted utilizing SPSS software (version 25.0, SPSS Inc. IL, USA), R software (version 4.2.2), and MSTATA software (www.mstata.com). The participants were divided into the TD-positive group and the TD-negative group. The optimal pair matching method in Propensity Score Matching (PSM) was used for 1:1 matching. Descriptive statistics were employed to depict the clinical and pathological characteristics of the participants and other baseline variables. Continuous variables conforming to a normal distribution were presented as mean ± standard deviation (SD), while non-normally distributed variables were represented as median (range). Categorical variables were described using ratios. The Mann-Whitney U test (for non-parametric distributions), chi-square test, or Fisher’s exact test (for categorical variables) were utilized to assess differences between the TD and non-TD groups before and after PSM. Single-factor and multiple-factor analyses of prognostic factors (ER cumulative incidence rate) were conducted using Cox regression models. The ER cumulative incidence rate was estimated using Kaplan-Meier curves, with comparisons made using the log-rank test. A p-value < 0.05 was considered statistically significant.

## Results

Among 11,278 patients with thyroid cancer, there were 235 cases of TD (2.08%). Among them, a total of 2,162 patients who met the inclusion criteria were included (TD-negative group: 2,056 cases, TD-positive group: 106 cases). TD was significantly associated with preablative stimulated thyroglobulin (Ps-Tg), cumulative RAI dose, number of LNM, maximum tumor diameter, T stage, N stage, extent of extrathyroidal extension, and solitary/multifocal primary lesions (**Table 1**).

**Table 1:** Patient demographics and baseline characteristics.

Characteristics	Original dataset			Matched dataset		
	TD negative	TD positive	p-value^1^	TD negative	TD positive	p-value^1^
Sample size, no.	2056	106		106	106	
Sex			0.375			0.769
Female	1,497 (72.8%)	73 (68.9%)		71 (67.0%)	73 (68.9%)	
Male	559 (27.2%)	33 (31.1%)		35 (33.0%)	33 (31.1%)	
Age, years	45.0 ± 11.7	45.6 ± 14.1	0.678	45.8 ± 12.9	45.6 ± 14.1	0.959
Maximum tumor diameter, cm	1.1 (0.7, 1.8)	1.7 (1.1, 2.2)	<0.001	1.0 (1.5, 2.5)	1.1 (1.7, 2.2)	0.696
T stage			0.003			0.966
T1a	376 (18.3%)	7 (6.6%)		6 (5.7%)	7 (6.6%)	
T1b	131 (6.4%)	10 (9.4%)		10 (9.4%)	10 (9.4%)	
T2	55 (2.7%)	5 (4.7%)		3 (2.8%)	5 (4.7%)	
T3a	7 (0.3%)	1 (0.9%)		0 (0.0%)	1 (0.9%)	
T3b	1,476 (71.8%)	81 (76.4%)		85 (80.2%)	81 (76.4%)	
T4a	10 (0.5%)	2 (1.9%)		2 (1.9%)	2 (1.9%)	
T4b	1 (0.1%)	0 (0.0%)		0 (0.0%)	0 (0.0%)	
N stage			<0.001			0.186
N0	237 (11.5%)	9 (8.5%)		5 (4.7%)	9 (8.5%)	
N1a	1,165 (56.7%)	37 (34.9%)		45 (42.5%)	37 (34.9%)	
N1b	633 (30.8%)	60 (56.6%)		56 (52.8%)	60 (56.6%)	
NX	21 (1.0%)	0 (0.0%)		0 (0.0%)	0 (0.0%)	
Number of LNM	3.0 (1.0, 7.0)	4.0 (2.0, 7.3)	0.026	5.0 (2.0, 9.0)	4.0 (2.0, 7.3)	0.914
Number of central LNM	2.0 (1.0, 5.0)	2.0 (1.0, 4.0)	0.035	2.0 (1.0, 5.0)	2.0 (1.0 4.0)	0.665
Number of lateral LNM	0.0 (0.0, 1.0)	1.5 (0.0, 4.3)	<0.001	1.0 (0.0, 5.0)	1.5 (0.0, 4.3)	0.719
Cumulative RAI dose, mCi.	100.0 (80.0, 150.0)	150.0 (100.0, 250.0)	<0.001	150.0 (100.0, 222.5)	150.0 (100.0, 250.0)	0.451
Ps-Tg, ng/mL	6.5 (2.2, 14.6)	13.0 (7.3, 31.9)	<0.001	9.2 (4.0, 30.4)	13.0 (7.3, 31.9)	0.778
Primary tumor multiplicity			0.003			0.444
Solitary	819 (39.8%)	27 (25.5%)		32 (30.2%)	27 (25.5%)	
Multiple	1,237 (60.2%)	79 (74.5%)		74 (69.8%)	79 (74.5%)	
Extrathyroidal extension			<0.001			0.981
Limited to the thyroid	562 (27.3%)	22 (20.8%)		19 (17.9%)	22 (20.8%)	
With minimal extrathyroidal extension	1,483 (72.1%)	82 (77.4%)		85 (80.2%)	82 (77.4%)	
Beyond the thyroid capsule	11 (0.5%)	2 (1.9%)		2 (1.9%)	2 (1.9%)	

^1^Pearson’s Chi-squared test; Welch Two Sample t-test; Wilcoxon rank sum test; Fisher’s exact test.

RAI, Radioiodine; TD, Tumor deposits; Ps-Tg, Preablative stimulated thyroglobulin; LNM, lymph node metastases.

### Relationship between TD and clinicopathologic characteristics

After PSM, 106 TD-positive patients were matched with 106 TD-negative patients. There were no statistically significant differences between the two groups in terms of gender, age, T stage, N stage, extent of extrathyroidal extension, cumulative RAI dose, Ps-Tg, number of LNM, solitary/multifocal primary lesions, and maximum tumor diameter. The distribution of baseline covariates was adequately balanced in the propensity score-matched dataset (**Table 1**).

### Prognostic analysis

**Figure 2:**
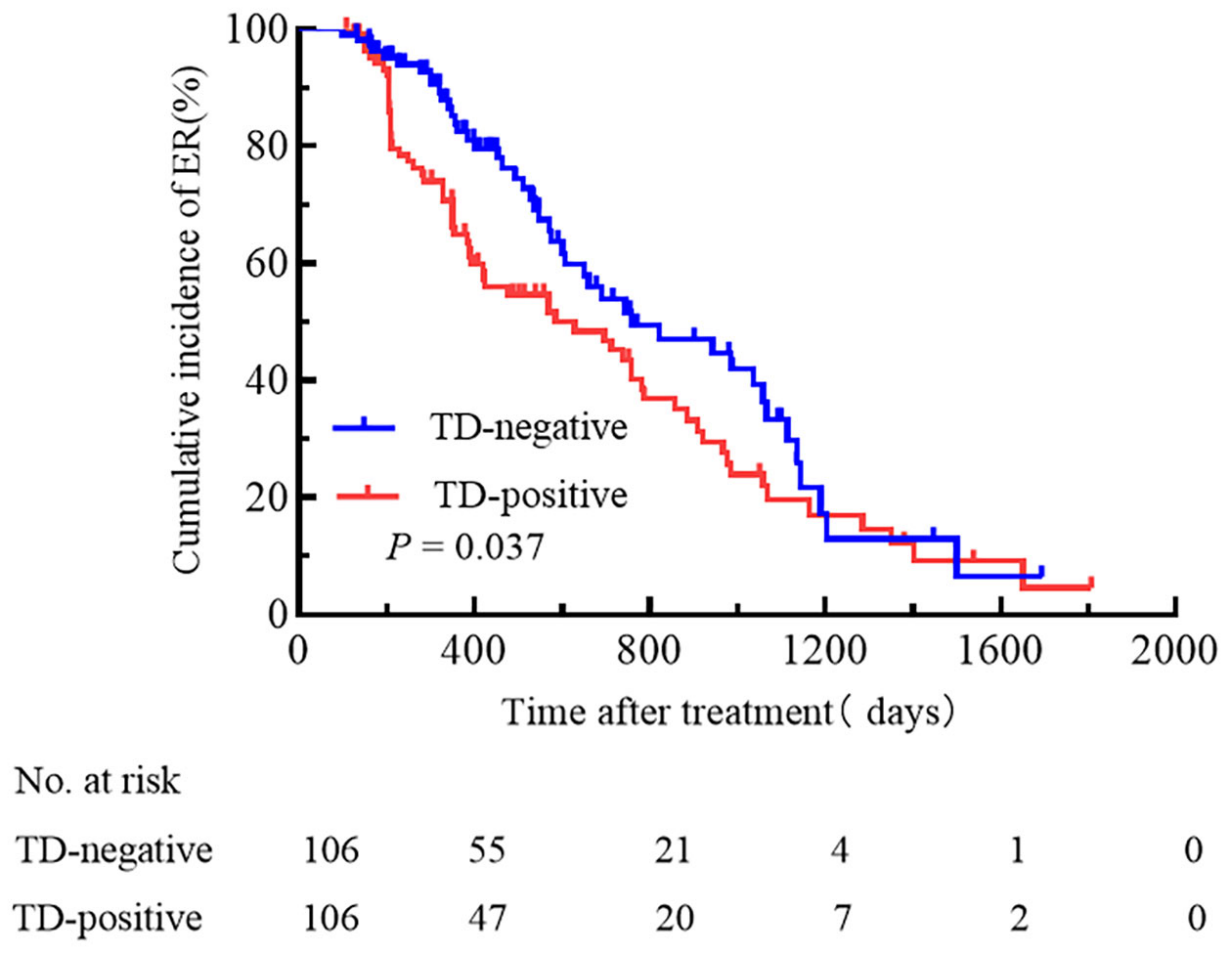
A comparison of the cumulative incidence rate of ER between TD-positive and TD-negative patients.

According to the single-factor analysis, the following 8 clinical and pathological characteristics were significantly associated with treatment efficacy in all enrolled patients: age, maximum tumor diameter, pN stage, presence of TD, cumulative RAI dose, Ps-Tg, the ATA initial risk stratification system, and TNM stage (**Table 2**). In the multivariable Cox proportional hazards model analysis, gender, presence of TD, the ATA initial risk stratification system, and TNM stage were identified as independent prognostic factors (**Table 2**). The cumulative incidence rate of ER in all enrolled patients was 47.6%, with 101 patients experiencing ER during the follow-up period. The cumulative incidence rate of ER was 37.7% for the TD-positive group and 57.5% for the TD-negative group. Kaplan-Meier curves were used to depict the long-term prognosis of the TD-negative and TD-positive groups. In all patients, there was a significant difference in the curves between the TD-positive group and the TD-negative group in terms of the cumulative incidence rate of ER (HR 1.49, 95% CI 1.02-2.17, p = 0.037, **Figure 2**).

**Table 2:** Univariate and multivariate analysis of influencing factors (Cox regression).

Characteristic	Univariable		Multivariable		
	N	p-value	HR^1^	95% CI^1^	p-value
Sex		0.033			0.024
Female	144		Reference		
Male	68		1.66	1.07, 2.58	0.024
Age, years	212	0.475			
Primary tumor multiplicity		0.584			
Solitary	59				
Multiple	153				
Maximum tumor diameter, cm	212	0.011	1.08	0.92, 1.28	0.328
Extrathyroidal extension		0.057			
Limited to the thyroid	41				
With minimal extrathyroidal extension	167	0.073			
Beyond the thyroid capsule	4	0.839			
T stage		0.063			
T1	33				
T2	8	0.581			
T3	167	0.623			
T4	4	0.314			
N stage		0.028			0.576
N0	14		Reference		
N1a	82	0.243	0.89	0.34, 2.37	0.824
N1b	116	0.011	0.78	0.48, 1.25	0.299
Number of LNM	212	0.371			
Number of central LNM	212	0.810			
Number of lateral LNM	212	0.181			
TD		0.039			0.024
Absent	106		Reference		
Present	106	0.039	0.24	0.68, 0.83	0.024
Cumulative RAI dose, mCi	212	0.001	1.00	1.00, 1.00	0.306
Ps-Tg, ng/mL	212	<0.001	1.00	1.00, 1.00	0.503
Risk of structural disease recurrence		0.003			0.034
Low risk	71		Reference		
Intermediate risk	91	0.517	0.44	0.21, 0.94	0.033
High risk	50	0.003	0.41	0.20, 0.81	0.011
TNM staging		0.001			0.010
I	133		Reference		
II	79	0.001	0.57	0.37, 0.88	0.010

^1^HR, Hazard Ratio; CI, Confidence Interval.

**Figure 3:**
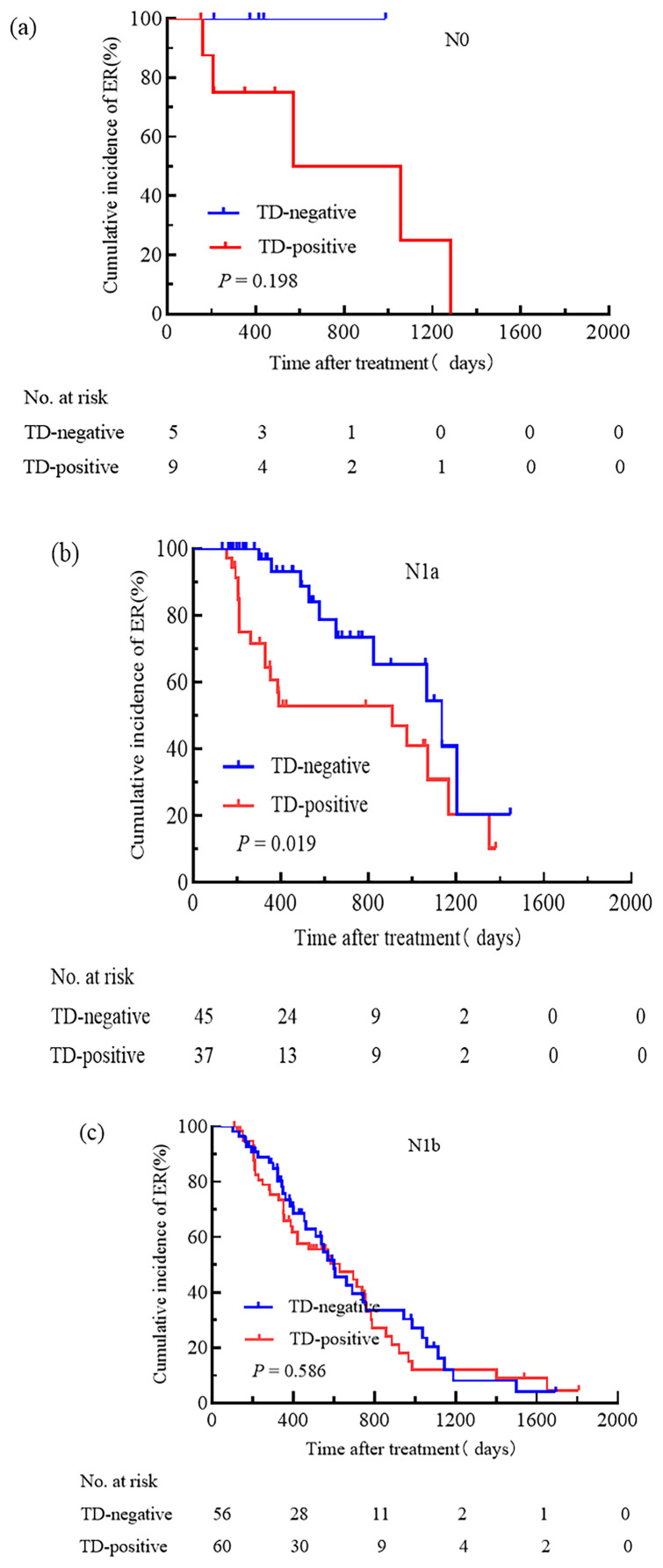
Comparison of the cumulative incidence rate of ER in TD-negative and TD-positive patients across different N stages. N0 **(a)**, N1a **(b)**, and N1b **(c)** stages for TD-negative and TD-positive groups.

**Figure 4:**
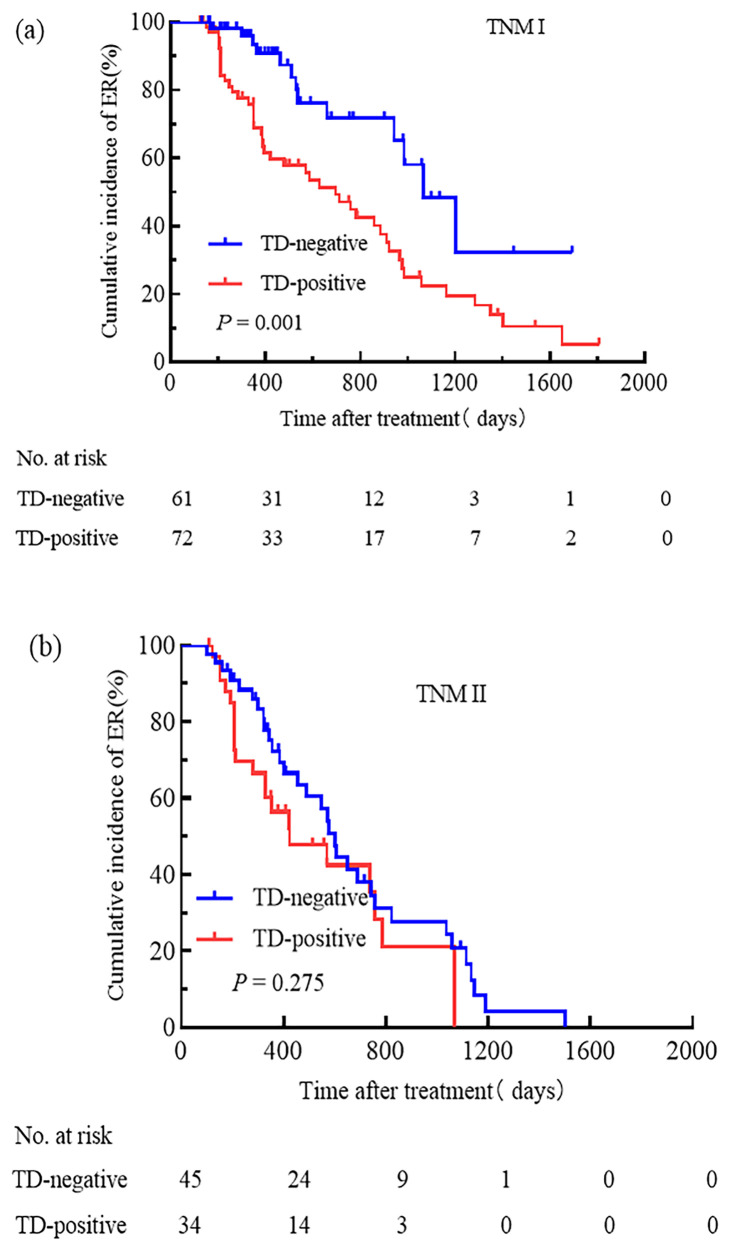
Comparison of the cumulative incidence rate of ER in TD-negative and TD-positive patients across different TNM stages. TNM stage I **(a)** and TNM stage II **(b)** for TD-negative and TD-positive groups.

**Figure 5:**
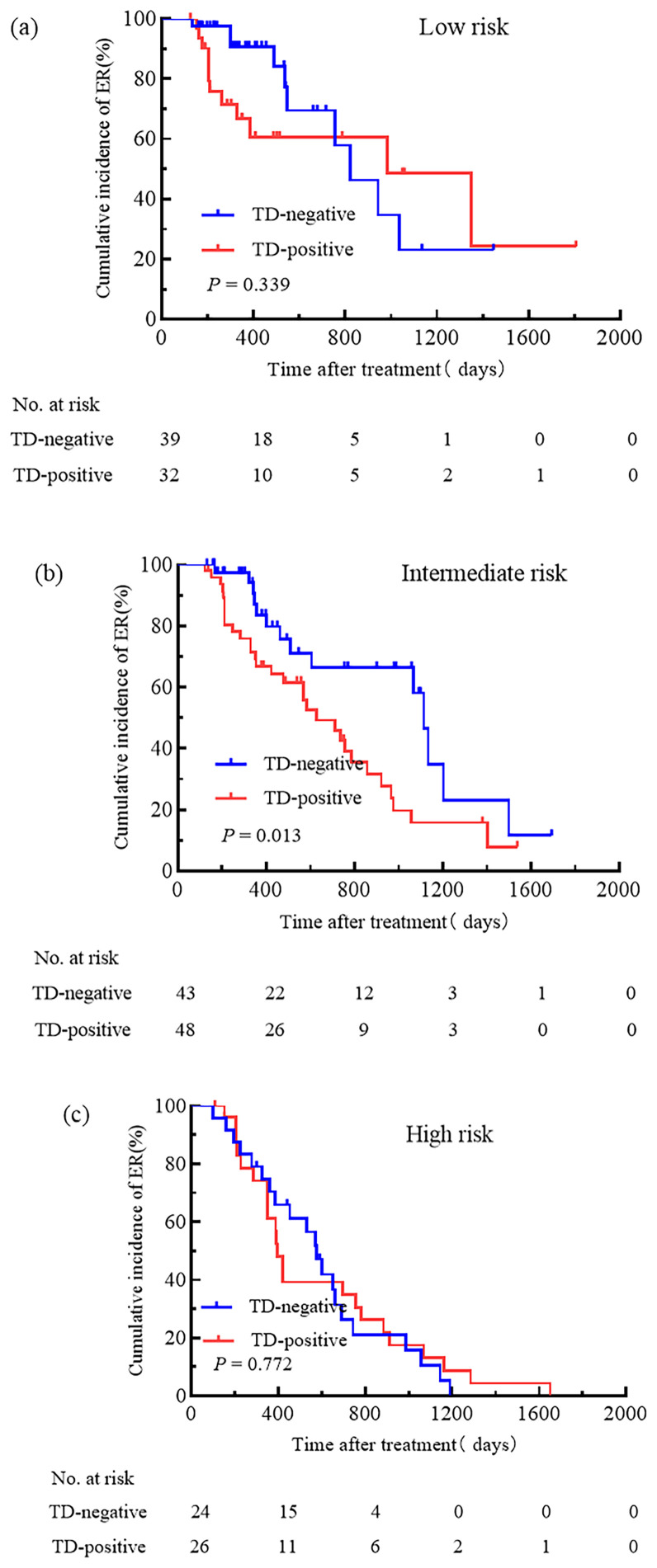
Comparison of the cumulative incidence rate of ER in TD-negative and TD-positive patients across different initial risk stratification. Low-risk **(a)**, Intermediate-risk **(b)**, and High-risk **(c)** patients for TD-Negative and TD-Positive groups.

We attempted to compare the relationships between pN stage, TNM stage, the ATA initial risk stratification system, and TD. Patients were grouped based on pN stage, TNM stage, and the ATA initial risk stratification system. The cumulative incidence rate of ER for pN0 patients with and without TD was 44.4% and 100.0%, respectively (**Figure 3a**); for pN1a patients with and without TD, the cumulative incidence rate of ER was 48.6% and 77.8% (**Figure 3b**); for pN1b patients with and without TD, the cumulative incidence rate of ER was 31.7% and 37.5% (**Figure 3c**); for patients with TNM stage I, the cumulative incidence rate of ER for TD-positive and TD-negative patients was 37.5% and 78.7%, respectively (**Figure 4a**); for patients with TNM stage II the cumulative incidence rate of ER for TD-positive and TD-negative patients was 28.9% and 38.2%, respectively (**Figure 4b**). In low-risk patients, the cumulative incidence rate of ER for TD-positive and TD-negative patients was 62.5% and 74.3%, respectively (**Figure 5a**); in intermediate-risk patients, the cumulative incidence rate of ER for TD-positive and TD-negative groups was 35.4% and 67.4% (**Figure 5b**); in high-risk patients, the cumulative incidence rate of ER for TD-positive and TD-negative patients was 11.5% and 12.5% (**Figure 5c**). Statistical differences were found between TD-positive and TD-negative groups in pN1a stage, TNM stage I, and intermediate-risk patients (with P values of 0.019, 0.001, and 0.013, respectively, as shown in **Figures 3b**, **4a**, **5b**).

**Figure 6:**
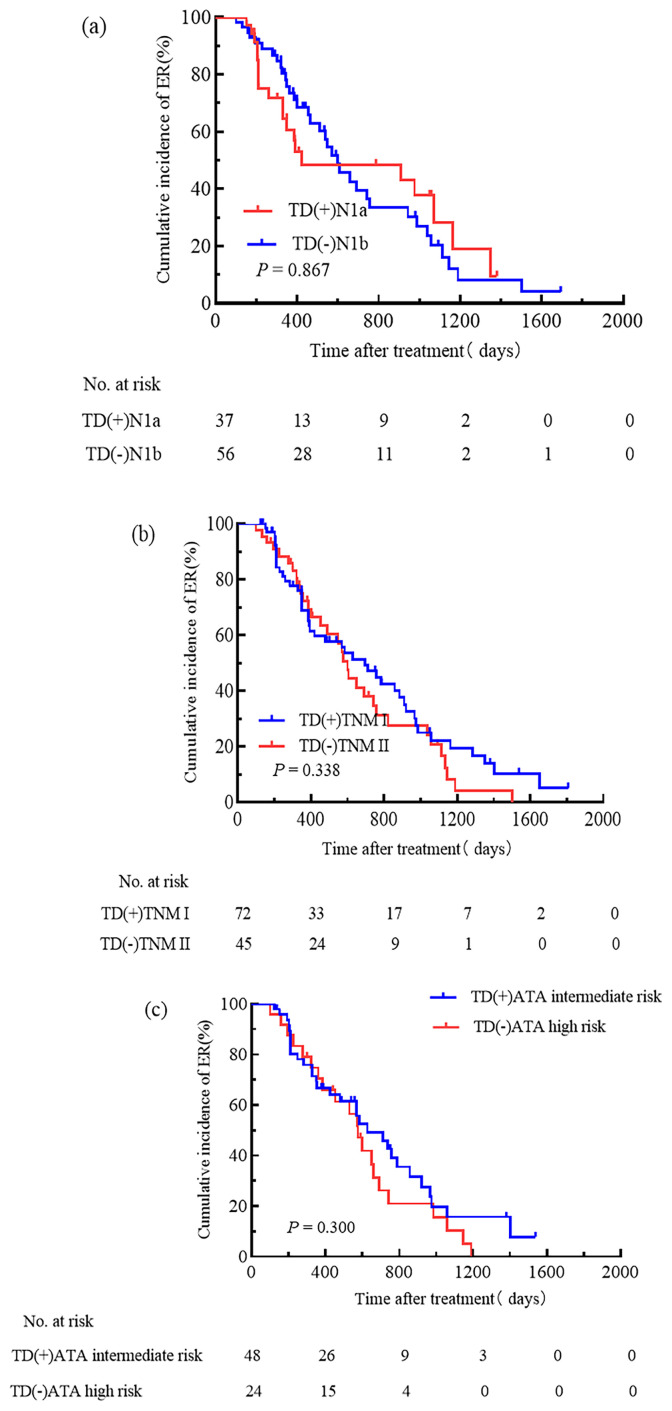
Incorporating TD into N stage, TNM staging, and the ATA initial risk stratification system. Cumulative incidence rate of ER was similar between TD-positive patients in N1a stage and TD-negative patients in N1b stage **(a)**; TD-positive patients in TNM stage I and TD-negative patients in TNM stage II had similar cumulative incidence rate of ER **(b)**; TD-positive intermediate-risk patients and TD-negative high-risk patients showed similar cumulative incidence rate of ER **(c)**.

Comparing TD-positive patients in N1a stage with TD-negative patients in N1b stage, the cumulative incidence rate of ER was similar (32.6% vs. 42.6%, HR 0.95, 95% CI 0.55–1.66, P = 0.867, **Figure 6a**). The cumulative incidence rate of ER was similar between TD-positive patients in TNM stage I and TD-negative patients in TNM stage II (37.5% vs. 28.9%, HR 0.79, 95% CI 0.50–1.28, P = 0.338, **Figure 6b**). There was no significant difference in the cumulative incidence rate of ER between intermediate-risk TD-positive patients and high-risk TD-negative patients (35.4% vs. 12.5%, HR 0.73, 95% CI 0.41–1.32, P = 0.300, **Figure 6c**).

## Discussion

In this study, we investigated the clinical and pathological significance of TD, confirming its negative impact in patients with DTC undergoing RAI treatment. Through univariate and multivariate analysis, we indicated that the prognosis of TD patients was poorer compared to non-TD patients. The prognostic impact was confirmed across N stage, TNM staging, and the ATA initial risk stratification system.

Previous studies have reported that the occurrence of TD is associated with various clinical and pathological factors. Xie et al. (11) demonstrated a positive correlation between TD and the depth of invasion as well as the presence of LNM. Some other studies have reported a correlation between tumor size and TD (12), but the threshold is not yet clear. The results of this study showed that the incidence rate of TD was 2.08%. Among all DTC cases undergoing RAI treatment, the formation of TD was closely associated with higher levels of Ps-Tg, cumulative RAI dose, number of LNM, higher T stage, N stage, larger maximum tumor diameter, greater extent of extrathyroidal extension, and multifocality of the primary lesion. This suggest that TD positivity was closely related to the malignancy and progression of the tumor.

To analyze the prognostic value of TD in DTC patients, we employed PSM analysis. By eliminating potential factors that could influence the results, we selected 106 TD-negative and 106 TD-positive DTC patients for prognostic analysis. The study results indicated that older age, larger tumor diameter, higher pN stage, cumulative RAI dose, Ps-Tg levels, the ATA initial risk stratification, TNM staging, and the presence of TD were associated with poor prognosis. Multifactor analysis further confirmed that TD was a significant predictive factor for treatment efficacy. The Kaplan-Meier survival curve showed that in the PSM cohort, the cumulative incidence rate of ER in TD-positive patients was significantly lower than in TD-negative patients. Therefore, how to stage the postoperative pathology in the presence of these distinctive nodules warrants consideration regarding whether they should be included in T stage, N stage, or other staging systems.

Currently, the AJCC staging system for colorectal cancer has included mesenteric TD in the N stage, defining it as stage N1c, to better guide postoperative treatment. Previous researchers have proposed revised versions of the TNM staging recommendations for different tumors. Anup et al. (13) suggested that TD may be more suitable as a form of serosal invasion. They included pT1–3 class TD in gastric cancer as pT4a class TD cancer. Chen et al. (14) are more inclined to include TD in the pN category for colorectal cancer and propose that, except for N3b patients, the presence of TDs would elevate the pN staging. Hua et al. (15) found that the prognosis of TD-positive patients is comparable to N2-stage patients who are TD-negative. Therefore, they suggest categorizing TD-positive patients in pancreatic cancer as stage III.

We separately investigated the impact of TD on N stage, TNM staging, and the ATA initial risk stratification system. We conducted a prognosis analysis on patients with TD and those without TD in stages N0, N1a, and N1b. The study results indicated that in the pN1a subgroup, the presence of TD was significantly associated with a poor prognosis. Additionally, the cumulative incidence rate of ER in patients with TD in stage pN1a was similar to that of patients without TD in stage pN1b. However, there was no significant difference between patients with or without TD in stages pN0 and pN1b. This differed from the findings of Hua et al. (15). Upon comparison, it was found that the two studies had different research methods: we analyzed the differences in the presence or absence of TD in different N stages, whereas the latter considered TD positivity as a whole and compared it with TD negativity across different N stages. In the TNM staging, we found a significant correlation between the presence of TD and poor prognosis in TNM stage I, with the therapeutic outcomes of TD-positive patients in TNM stage I being similar to those of TD-negative patients in TNM stage II. However, in TNM stage II, the difference in prognosis between TD-positive and TD-negative cases was not significant. This was similar to the study by Belt et al. (16), where they compared the survival of TD-positive patients in stage II with TD-negative patients in stage III of colorectal cancer. They suggested that all colorectal cancer patients with TD involving the mesentery or perirectal tissues should be classified as stage III. In addition, in the ATA initial risk stratification system, we found that the presence of TD was associated with a poor prognosis in intermediate-risk patients. However, there were no significant differences between patients with or without TD in low-risk and high-risk groups. The cumulative incidence rate of ER in intermediate-risk patients with TD was similar to that of high-risk patients without TD. Therefore, we propose a plan: for patients with TD in stage N1a, they should be classified under stage N1b; patients with TD in TNM stage I should be classified under TNM stage II, and TD patients classified as intermediate-risk should be included in the high-risk group.

There are still some limitations in this study. Firstly, our study focused on patients receiving RAI treatment, which may not fully represent the distribution in the population of DTC patients. Secondly, the number of patients in stage pN0 was small (13 cases), which might limit the comprehensiveness of the prognosis analysis for N0 stage patients. Furthermore, this was a single-center retrospective study, which was limited by the typical drawbacks associated with retrospective analyses. Further confirmation in multicenter studies would be beneficial in determining the value of TD and enhancing the accuracy of the DTC staging system.

In conclusion, the occurrence of TD was associated with various clinicopathological factors, and TD served as an independent prognostic factor for postoperative RAI treatment in DTC patients. We have proposed a clinically useful approach by incorporating TD into the TNM staging system and the ATA initial risk stratification system: categorizing N1a patients with TD as N1b stage; TNM stage I patients as TNM stage II; and intermediate-risk patients as high-risk.

## Data Availability

The original contributions presented in the study are included in the article/supplementary material. Further inquiries can be directed to the corresponding authors.
